# Visceral Adipose Tissue Inflammation and Vascular Complications in a Rat Model with Severe Dyslipidemia: Sex Differences and PAI-1 Tissue Involvement

**DOI:** 10.3390/biom15010019

**Published:** 2024-12-27

**Authors:** Irena Markova, Martina Hüttl, Natalie Gayova, Denisa Miklankova, Kristyna Cerna, Martina Kavanova, Petra Skaroupkova, Sona Cacanyiova, Hana Malinska

**Affiliations:** 1 Centre for Experimental Medicine, Institute for Clinical and Experimental Medicine, 140 21 Prague, Czech Republic; irma@ikem.cz (I.M.); mabw@ikem.cz (M.H.); gayova.natalie@gmail.com (N.G.); mild@ikem.cz (D.M.); ceak@ikem.cz (K.C.); pesp@ikem.cz (P.S.); 2 Department of Laboratory Methods, Institute for Clinical and Experimental Medicine, 140 21 Prague, Czech Republic; kavm@ikem.cz; 3 Centre of Experimental Medicine, Institute of Normal and Pathological Physiology, Slovak Academy of Sciences, 841 04 Bratislava, Slovakia; sona.cacanyiova@savba.sk

**Keywords:** visceral adipose tissue, perivascular adipose tissue, inflammation, hypertriglyceridemia, plasminogen activator inhibitor-1, cardiovascular disease

## Abstract

We investigated the sex-dependent effects of inflammatory responses in visceral adipose tissue (VAT) and perivascular adipose tissue (PVAT), as well as hematological status, in relation to cardiovascular disorders associated with prediabetes. Using male and female hereditary hypertriglyceridemic (HHTg) rats—a nonobese prediabetic model featuring dyslipidemia, hepatic steatosis, and insulin resistance—we found that HHTg females exhibited more pronounced hypertriglyceridemia than males, while HHTg males had higher non-fasting glucose levels. Additionally, HHTg females had higher platelet counts, larger platelet volumes, and lower antithrombin inhibitory activity. Regarding low-grade chronic inflammation, HHTg males exhibited increased serum leptin and leukocyte levels, while females had increased serum interleukin-6 (IL-6). Both sexes had increased circulating plasminogen activator inhibitor-1 (PAI-1), higher PAI-1 gene expression in VAT and PVAT, and elevated intercellular adhesion molecule-1 (ICAM-1) gene expression in the aorta, contributing to endothelial dysfunction in the HHTg strain. However, HHTg females had lower tumor necrosis factor alpha (TNFα) gene expression in the aorta. Severe dyslipidemia in this prediabetic model was associated with hypercoagulation and low-grade chronic inflammation. The increase in PAI-1 expression in both VAT and PVAT seems to indicate a link between inflammation and vascular dysfunction. Despite the more pronounced dyslipidemia and procoagulation status in females, their milder inflammatory response may reflect an association between reduced cardiovascular damage and prediabetes.

## 1. Introduction

Cardiovascular disease complications, which account for roughly 50 to 70% of mortality in people with diabetes, typically develop during prediabetic and insulin-resistant states. Metabolic disorders such as insulin resistance, adipose tissue dysfunction, impaired lipid metabolism, and hepatic steatosis, all of which accompany prediabetes, increase cardiovascular damage and serve as independent cardiovascular risk factors [1]. These risks also tend to vary between men and women. The presence of risk factors for metabolic syndrome increases the risk of cardiovascular heart disease by 2.4 times in men and 5.9 times in women [2]. Individuals with prediabetes also have a hemostatic imbalance, which can contribute to the early development of diabetes and its associated vascular complications. Coagulation imbalance and impaired fibrinolysis have been shown to influence hemostatic alterations in metabolic syndrome and prediabetes [3].

Low-grade chronic inflammation, combined with severe dyslipidemia, can exacerbate both thrombophilic and vascular risks. Elevated circulating lipids and lipoproteins not only have a proatherogenic effect but also directly contribute to thrombotic conditions by interacting with hemostatic factors and platelets (PLTs) [4]. The increased metabolic activity of PLTs may exacerbate chronic inflammation. While the exact hematological changes associated with prediabetes are not fully understood, disturbances in lipid metabolism, hepatic steatosis, low-grade chronic inflammation, and insulin resistance are assumed to be significant contributors. Additionally, evidence suggests that ectopic fat deposits, including hepatic, epicardial, and perivascular fat, play a role in increasing cardiometabolic risk [5].

At the systemic level, visceral adipose tissue (VAT) dysfunction contributes to chronic inflammation [6]. The activation of inflammatory pathways, reflected in altered coagulation and fibrinolysis, depends on the interaction between different types of cells, such as PLTs, leukocytes, and endothelial cells. Adipokines like leptin, monocyte chemoattractant protein-1 (MCP-1), TNFα, and IL-6 regulate key metabolic processes, including glucose and lipid metabolism, as well as atherosclerosis. Plasminogen activator inhibitor-1 (PAI-1), a critical regulator of fibrinogenesis, has recently been implicated in various cellular processes beyond its well-established role in fibrinolysis [7]. Elevated PAI-1 levels are associated with several conditions, including metabolic syndrome, diabetes, insulin resistance, vascular thrombosis, and atherosclerosis [8]. At the local level, perivascular adipose tissue (PVAT), which surrounds blood vessels, plays a role in vascular pathology by exerting metabolic, inflammatory, and vasoactive effects. In fact, differences in the metabolic activity of PVAT and VAT in conjunction with the effects of PAI-1 are understood to contribute to variations in cardiovascular risk and disease between sexes. Recently, PAI-1 activity influenced by visceral fat accumulation was higher in women than in men, but serum level of estrogen could not explain the sex difference [7]. Additionally, the activity of PVAT and VAT could contribute to sex differences in relation to cardiovascular damage. However, the precise mechanisms are not clear.

To disentangle these associations, we investigated a number of sex-dependent metabolic, inflammatory, and hematological parameters in relation to cardiovascular damage using the hereditary hypertriglyceridemic (HHTg) rat—a prediabetic model featuring severe dyslipidemia and hepatic steatosis [9,10]. Our previous study of HHTg males showed that a combination of redox imbalance, inflammation, and altered NO bioavailability contributes to endothelial dysfunction [9]. Building on our previous findings [11], the present study reveals that HHTg female rats are more protected from vascular dysfunction than males. Our previous results also confirm that PVAT activity plays a significant role in preserving endothelial function and contractility [12].

## 2. Materials and Methods

### 2.1. Animals

All experiments were performed in agreement with the Animal Protection Law of the Czech Republic (311/1997), which complies with European Community Council recommendations (86/609/ECC) on the use of laboratory animals. Six-month-old male and female Wistar–Kyoto rats (WKY) were used as the control group. Six-month-old male (average BW 432 g) and female (average BW 257 g) HHTg rats (provided by the Institute for Clinical and Experimental Medicine, Prague, Czech Republic), a nonobese prediabetic model, served as the experimental group. This rat strain exhibits genetically determined hypertriglyceridemia, insulin resistance in peripheral tissue, and liver steatosis in the absence of obesity and fasting hyperglycemia. At the beginning of the study, the animals were randomized into four experimental groups (WKY male, WKY female, HHTg male, and HHTg female), with eight rats in each group (*n* = 8). The rats were housed under temperature- (22 °C) and humidity-controlled conditions following a 12 h/12 h light/dark cycle with free access to food (maintenance diet for rats and mice; Altromin, Germany, Lage) and drinking water. At the end of the experiment, the rats were euthanized in a postprandial state after light anesthesia (zoletil 5 mg/kg b.wt.). Aliquots of serum, plasma (EDTA, sodium citrate), and tissue samples were collected and stored at −80 °C for further analysis.

### 2.2. Analytical Methods

Serum levels of triglycerides, glucose, alanine aminotransferase (ALT), aspartate aminotransferase (AST), total cholesterol, and high-density lipoprotein (HDL) cholesterol were measured using commercially available kits (Erba Lachema, Brno, Czech Republic; Roche Diagnostics, Mannheim, Germany). Serum insulin and leptin concentrations were determined using the Rat Insulin ELISA kit and the Leptin Elisa kit (Mercodia AB, Sweden; Biovendor, Czech Republic). Serum levels of MCP-1, TNFα, PAI-1, IL-6, and high-sensitivity C-reactive protein (hsCRP) were measured using rat ELISA kits from MyBiosource (San Diego, CA, USA), eBioscience/Bender MedSystems (Austria, Vienna), and Alpha Diagnostics International (San Antonio, TX, USA).

To determine liver triglycerides and cholesterol levels, liver samples with an initial amount of 200 mg were powdered under liquid N_2_ and extracted in a chloroform/methanol solution. The organic phase was removed and evaporated under N_2_. The resulting pellet was then dissolved in isopropyl alcohol. The triglyceride and cholesterol content was determined by enzymatic assay (Erba-Lachema, Czech Republic, Brno) [13].

### 2.3. Hematological and Coagulation Parameters

Plasma hemoglobin (Hb) concentration was determined using a cyanate-free spectrometric method based on a reaction with sodium lauryl sulfate (SLS). Plasma fibrinogen concentration was monitored using the Clauss coagulation method to quantify the amount of clottable protein (Stago, Parsippany, NJ, USA). The inhibitory activity of antithrombin (AT) was quantitatively determined using a spectrophotometric method featuring a chromogenic substrate bound to p-nitroanilide (Stago, Parsippany, NJ, USA). The blood count was measured using a morphology line from Sysmex, consisting of a coating and staining machine and an analyzer to enable digital morphology. The Sysmex XN device provided a detailed analysis of blood picture parameters, including a five-part differential count and the fraction of immature granulocytes. PLT and red blood cell (RBC) counts were determined by impedance. Other parameters, including the differential leukocyte count, were measured by flow cytometry using a semiconductor laser (WDR channel on the Sysmex XN device). This method was successful in differentiating leukocytes into lymphocytes, monocytes, eosinophils, basophils, neutrophils, and immature granulocytes. Additionally, a blood smear was prepared from each sample and stained with Pappenheim panoptic staining (May–Grünwald and Giemsa–Romanowski). To confirm the accuracy of the PLT counts, the absence of PLT clusters in each sample was verified using an Olympus microscope at 500× magnification.

### 2.4. Relative mRNA Expression

Total RNA was isolated from the tissues using RNA Blue (Top-Bio, Czech Republic, Prague). RNA was quantified using Nanodrop One (Thermo Fisher Scientific, Waltmam, MA, USA) to determine yield and purity. Only samples with an A260/280 ratio of 1.9–2.1 and an A260/230 ratio 2.0–2.2 were utilized in further steps. Reverse transcription and quantitative real-time PCR analysis was performed using the TaqMan RNA-to-C_T_ 1-Step Kit, the TaqMan Gene Expression Assay, and the ViiA 7 Real-Time PCR System (all Thermo Fisher Scientific, MA, USA). Relative gene expression was calculated using the 2^−ΔΔCt^ method after normalizing against *Hprt1* as the internal reference. The results were run in triplicate.

### 2.5. Statistical Analysis

The statistical analysis was performed using StatSoft Statistica 14 (StatSoft CZ, Czech Republic, Prague). Two-way ANOVA was used to analyze the effects of sex and rat strain. All data analyzed followed a normal distribution according to the Shapiro–Wilk test. Fisher’s LSD post hoc test was used for variables showing evidence of sex-by-strain interactions. The test was adjusted for multiple comparisons to determine whether sex and strain would significantly influence metabolic and hematological parameters. Statistical significance was set at *p* < 0.05. All results are expressed as the mean ± SEM. Finally, Pearson correlation coefficients were calculated using regression analysis to evaluate the relationships between variables.

## 3. Results

### 3.1. Sex-Dependent Effects of Severe Dyslipidemia on Adiposity, Glucose, and Lipid Metabolism

**Figure 1:**
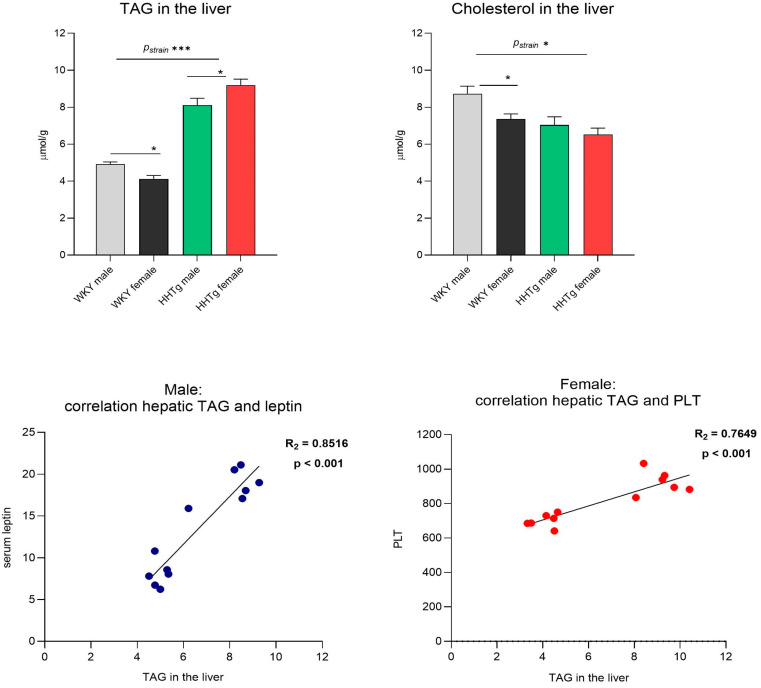
Sex-dependent effects of severe dyslipidemia on hepatic lipids correlated with Wistar–Kyoto control and prediabetic HHTg rats. Data are expressed as the mean ± SEM analyzed using two-way ANOVA and Fisher’s LSD post hoc test; TAG—triglycerides; PLT—platelets; * *p* < 0.05, *** *p* < 0.001.

Compared to the controls, the HHTg rats exhibited severe hypertriglyceridemia (*p*-_STRAIN_ < 0.001), decreased HDL cholesterol, and increased ectopic triglyceride (TAG) accumulation in the liver (*p*-_STRAIN_ < 0.001). HHTg females had higher serum TAG concentrations (*p* < 0.01) and higher hepatic TAG accumulation (*p* < 0.05) compared to age-matched HHTg males, which was associated with a significantly increased homeostatic model assessment of insulin resistance (HOMA-IR) index. Conversely, compared to the controls, the HHTg strain exhibited decreased serum and hepatic cholesterol concentrations (Table 1 and Figure 1). HHTg female rats had a significantly higher adiposity index (*p* < 0.01) than HHTg males, whereas HHTg males exhibited higher non-fasting glucose levels (*p* < 0.05). The HHTg rats also exhibited hyperinsulinemia, which was significantly higher in HHTg males compared to HHTg females. In addition, hepatic TAG accumulation were positively correlated with serum leptin levels (*p* < 0.05) in HHTg males and the PLT count (*p* < 0.01) in HHTg females (Figure 1).

### 3.2. Sex-Dependent Effects of Severe Dyslipidemia on Hematological and Coagulation Factors

Severe dyslipidemia was associated with alterations in hematological and coagulation parameters (Table 2). Compared to the WKY controls, the HHTg rats exhibited lower AT inhibitory activity and a higher PLT count and volume, leading to an increased plateletcrit (PCT). The fibrinogen and factor VIII levels, however, were not elevated in the HHTg rats. We observed significant sex differences in coagulation parameters: Compared to age-matched HHTg males, HHTg females had a significantly higher PLT count and volume (*p* < 0.01) and lower AT inhibitory activity (*p* < 0.01), potentially contributing to greater cardiovascular damage (Table 2). A blood smear confirmed the absence of any PLT clusters or abnormal pathological elements.

Table 2 shows the changes in the RBC parameters, which are characterized by significant sex differences. Compared to the control strain, the HHTg rats had higher values for the mean corpuscular volume (MCV), mean corpuscular hemoglobin concentration (MCHC), and mean concentration of hemoglobin (MCH), while maintaining similar hematocrit (HCT) values. The similarity in hematocrit values between the two strains can be attributed to the presence of fewer but larger erythrocytes in the HHTg rats.

### 3.3. Sex-Dependent Effects of Severe Dyslipidemia on Systemic Inflammation

**Figure 2:**
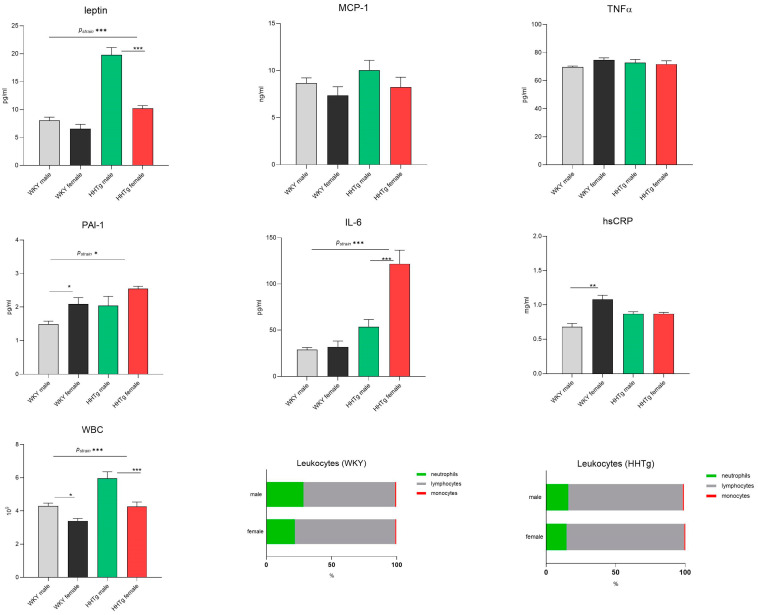
Sex-dependent effects of severe dyslipidemia on systemic inflammatory markers in Wistar–Kyoto control and prediabetic HHTg rats. Data are expressed as the mean ± SEM analyzed using two-way ANOVA and Fisher’s LSD post hoc test; MCP-1—monocyte chemoattractant protein-1; TNFα—tumor necrosis factor alpha; PAI-1—plasminogen activator inhibitor-1; IL-6—interleukin-6; hsCRP—high-sensitivity C-reactive protein; WBC—white blood cells. * *p* < 0.05, ** *p* < 0.01, *** *p* < 0.001.

As shown in Figure 2, severe hypertriglyceridemia in the HHTg rats was associated with low-grade chronic inflammation, characterized by significantly elevated serum levels of leptin, IL-6, and PAI-1. Inflammatory parameters differed between the sexes: HHTg males had higher leptin levels (*p* < 0.001), while females exhibited higher levels of interleukin-6 (IL-6) (*p* < 0.001). No significant differences in MCP-1, hsCRP, or TNFα between the sexes and strains were detected, except for serum hsCRP in the control group. In the HHTg rats, the activation of white blood cells (WBCs), along with a 15% increase in lymphocytes, contributed to low-grade chronic inflammation (Figure 2) and a deterioration in coagulation and cardiovascular functions.

### 3.4. Sex-Dependent Effects of Severe Dyslipidemia on PAI-1 Gene Expression in Different Tissues

**Figure 3:**
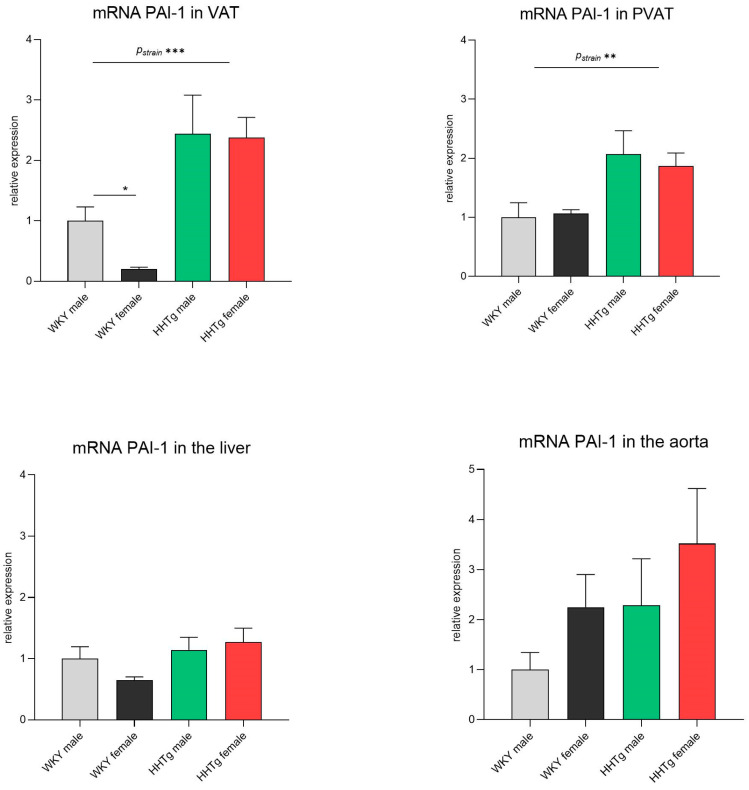
Sex-dependent effects of severe dyslipidemia on relative PAI-1 gene expression in different tissues in Wistar–Kyoto control and prediabetic HHTg rats. Data are expressed as the mean ± SEM analyzed using two-way ANOVA and Fisher’s LSD post hoc test; PAI-1—plasminogen activator inhibitor-1; VAT—visceral adipose tissue; PVAT—perivascular adipose tissue. * *p* < 0.05, ** *p* < 0.01, *** *p* < 0.001.

Higher serum PAI-1 concentrations in the HHTg rats were compared with the relative gene expression of PAI-1 in various tissues (Figure 3). While PAI-1 mRNA levels in the liver showed no significant differences, the HHTg rat strain exhibited significantly increased relative gene expression in both VAT (epididymal in males and perimetrial in females) and PVAT (Figure 3). No significant changes in PAI-1 mRNA expression were observed in the aorta.

### 3.5. Sex-Dependent Effects of Severe Dyslipidemia on the Gene Expression of Inflammatory Markers in Visceral and Perivascular Adipose Tissues

**Figure 4:**
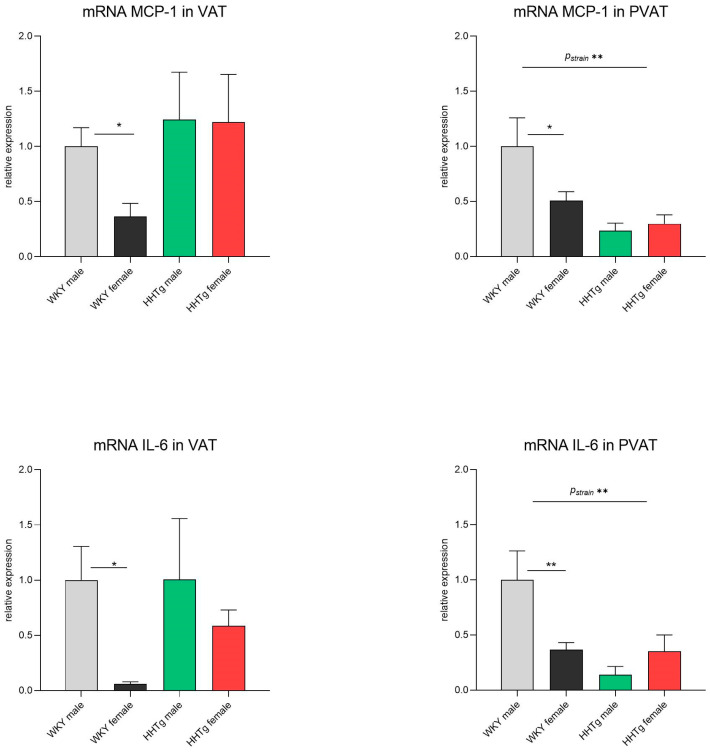
Sex-dependent effects of severe dyslipidemia on the relative gene expression of inflammatory markers in visceral and perivascular adipose tissues in Wistar–Kyoto control and prediabetic HHTg rats. Data are expressed as the mean ± SEM analyzed using two-way ANOVA and Fisher’s LSD post hoc test; MCP-1—monocyte chemoattractant protein-1; IL-6—interleukin-6; VAT—visceral adipose tissue; PVAT—perivascular adipose tissue. * *p* < 0.05, ** *p* < 0.01.

As shown in Figure 4, the prediabetic HHTg rats exhibited significantly decreased relative mRNA expression of the inflammatory markers MCP-1 and IL-6 in PVAT compared to the aged-matched controls. However, no significant differences in these markers were observed between the rat strains in VAT. In the WKY controls, females had a significantly decreased relative mRNA expression of MCP-1 and IL-6 in both VAT and PVAT (Figure 4). Notably, these effects were not observed in the HHTg rats.

### 3.6. Sex-Dependent Effects of Severe Dyslipidemia on the Gene Expression of Vascular and Inflammatory Markers in the Aorta

**Figure 5:**
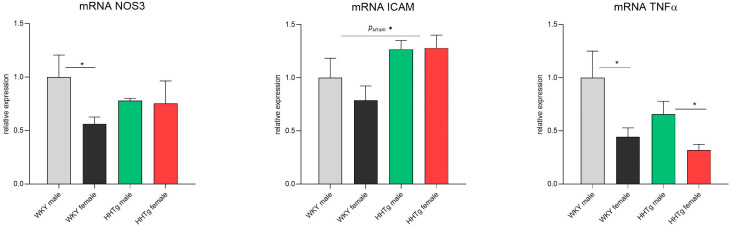
Sex-dependent effects of severe dyslipidemia on the relative gene expression of vascular and inflammatory markers in the aorta in Wistar–Kyoto control and prediabetic HHTg rats. Data are expressed as the mean ± SEM analyzed using two-way ANOVA and Fisher’s LSD post hoc test; NOS—nitric oxide synthase; ICAM-1—intercellular adhesion molecule-1; TNFα—tumor necrosis factor alpha. * *p* < 0.05.

As shown in Figure 5, the HHTg rat strain exhibited significantly increased relative gene expression of intercellular adhesion molecule-1 (ICAM-1) in the aorta compared to the controls. However, no significant differences were observed in the relative gene expression of NOS3 or TNFα between the two strains. In the WKY female rats, decreased mRNA expression of NOS3 was noted, but this effect was not confirmed in the HHTg rat strain. For the inflammatory factor TNFα, the females of both rat strains showed significantly reduced relative mRNA expression compared to age-matched males (Figure 5).

## 4. Discussion

Dyslipidemia, often seen in individuals with metabolic syndrome and prediabetes, not only plays a key role in cardiovascular impairment but also increases the risk of thrombosis. Circulating lipids and lipoproteins interact with hemostatic factors, modifying gene expression and PLT activation [3]. Although hypercholesterolemia and low-density lipoproteins (LDLs) enhance PLT hyperaggregability, hypertriglyceridemia and dysfunctional or reduced HDL particles also affect thrombocyte metabolism. Patients with hypertriglyceridemia display a prolonged blood clot lysis time, consistent with the finding that fibrinolysis is attenuated in patients with dyslipidemia [14]. In our study, severe hypertriglyceridemia was associated with lower AT inhibitory activity and a higher PLT count and volume. Larger PLTs are metabolically more active and, in addition to their role in fibrinogenesis, have the potential to promote chronic inflammation. Dyslipidemia is associated with an increased PLT count, PLT activity, hypercoagulability, and impaired fibrinolysis [4]. Deng et al. found that hyperlipidemic rats placed on a high-fat diet exhibited aggravated fibrinolysis and increased PLT aggregation [15].

Increased TAG and TAG-rich lipoproteins can directly activate PLTs, enhancing their responsiveness [16]. Conversely, HDL particles mediate antithrombotic effects by decreasing tissue factor (TF) expression. Additionally, a reduction in HDL particles can upregulate thrombin generation. HDL also prevents the self-association of von Willebrand factor (vWf) and its absorption onto vessel walls, inhibiting PLT adhesion and subsequent activation [17]. Our results are consistent with studies showing that HDL cholesterol is negatively associated with the PLT count in humans [18], and that HDL infusion reduces PLT counts in mice [19]. In our study, severe hypertriglyceridemia in HHTg rats was associated with impaired fibrinolysis, which may indicate a link between dyslipidemia and an increased risk of thrombosis. Lower AT inhibitory activity and higher PAI-1 levels may contribute to this impaired fibrinolysis. However, we found no increase in fibrinogen, vWf, or factor VIII concentrations between the two strains, which supports the idea that the relationship between fibrinogen and features of metabolic syndrome is weaker compared to that with other hemostatic factors such as PAI-1 [3].

Oxidized lipids and lipoperoxidation play an important role in lipid-induced PLT aggregation. Isoprostanes, which are oxidation metabolites of arachidonic acid, contribute to PLT aggregation by activating the thromboxane receptor. Oxidized LDL (oxLDL) induces PLT activation through CD36 and increases TF expression in macrophages and vascular smooth muscle cells [4]. Additionally, lysophosphatidic acid, a component of oxidized lipoprotein, increases TF expression and activity in vascular smooth muscle cells derived from rat aortas [20].

In our study, fatty-liver-associated hepatic lipid accumulation led to an increase in serum PAI-1 levels. These results are in agreement with a study involving human hepatoma cells, which found that TAG-rich very-low-density lipoproteins (VLDLs) stimulate PAI-1 secretion [21]. Furthermore, in hepatocytes, where lipids are incorporated into lipoproteins, the plasminogen activator inhibitor-1–tissue plasminogen activator (PAI-1–tPA) regulatory network influences the degree of fibrinolysis impairment. Lipid-overloaded hepatocytes lead to increased PAI-1 levels, which in turn stimulate tPA synthesis [22]. Experimental evidence has shown that VLDLs promote clotting via the procoagulant effect of phospholipids on lipoprotein particle surfaces, where oxidized phosphatidylcholines are able to enhance thrombin generation [23]. Therefore, hepatic lipid accumulation can impair fibrinolysis through alterations in VLDL particles without changing PAI-1 gene expression in the liver. Mouse studies highlight the causal roles played by PAI-1 and fibrinogen in regulating lipoprotein metabolism [24,25], influencing VLDL assembly and secretion through their effects on tPA, and potentially promoting postprandial lipoprotein lipase (LPL) activity [26].

Both severe dyslipidemia and insulin resistance contribute to impaired PLT function and altered metabolic activity. Insulin resistance, a critical pathogenic factor, plays a crucial role in PLT hyperactivation, linking it to vascular disease [27]. In agreement with our previous results, HHTg rats have been shown to exhibit both peripheral and hepatic insulin resistance in association with liver steatosis [28]. Under conditions of insulin resistance, the inhibitory effects on PLTs are attenuated, potentially contributing to their metabolic activation and hypercoagulation. Alterations in the plasma adipokine profile can also influence the effects of insulin on PLTs. In particular, resistin, leptin, and PAI-1 induce insulin resistance by interfering with the expression of insulin receptor substrate-1 (IRS-1), negatively impacting insulin signaling in PLTs [29]. Insulin typically exerts inhibitory effects on PLT aggregation [27]; thus, hyperinsulinemia, as observed in our study, can contribute to PLT hypercoagulation and hyperactivation.

The alterations in RBCs and hematocrit observed in this study are probably due to hyperinsulinemia and associated insulin resistance, where insulin plays a role in regulating erythropoiesis [30]. In patients with type 2 diabetes, RBCs are more susceptible to aggregation. Additionally, hyperglycemia and hyperlipidemia can cause membrane lipoperoxidation and increase the osmotic fragility of RBCs [31]. Chronic inflammation associated with prediabetes can also affect erythropoiesis, resulting in a decreased RBC half-life and deformability, thereby increasing the red blood cell distribution width (RDW).

Several adipokines and inflammatory cytokines can disrupt hemostatic balance, coagulation, and PLT function. Our study found that HHTg rats exhibited low-grade chronic inflammation, with prediabetic HHTg rats exhibiting higher circulating levels of leptin, IL-6, PAI-1, and leukocytes. Prediabetic HHTg males displayed more pronounced inflammation than females, with sex-specific differences in individual inflammatory markers. HHTg males had increased leptin and leukocyte levels, while females had elevated IL-6 levels. PAI-1, known for its antifibrotic effects and influence on vascular function, insulin resistance, and local inflammation, can significantly contribute to a procoagulant state. High levels of PAI-1, which promote thrombosis (by inhibiting fibrinolysis) and inflammation, are associated with the development of macrovascular complications. Elevated circulating PAI-1 levels have been observed in patients with metabolic syndrome and type 2 diabetes [8], as well as in animal models of diabetes [7].

Beyond PAI-1, several other adipokines contribute to a prothrombotic state. For instance, leptin influences PLT function and coagulation balance, making elevated leptin levels an independent cardiovascular risk factor [16]. Leptin also enhances vascular thrombosis by inducing PLT adhesion, activation, and aggregation in mice [32]. In human endothelial cells, leptin increases PAI-1 levels, leading to a procoagulant state [33]. Although higher leptin levels are typically observed in women (due to a higher amount of subcutaneously adipose tissue rather than direct sex hormonal effects), surprisingly, our study found higher leptin levels in HHTg males compared to HHTg females, potentially increasing cardiovascular impairment in HHTg males. Higher leptin levels in HHTg males may not directly correlate with visceral adipose tissue mass, but rather with insulin and leptin resistance. Elevated leptin and PAI-1 levels exacerbate insulin resistance, interfere with IRS-1, and negatively impact insulin signaling in PLT membranes [27]. Adipose tissue plays a direct role in the prothrombotic tendency of patients with metabolic syndrome and diabetes [5]. Systemic inflammation impairs hemostatic balance by stimulating PAI-1 synthesis in adipocytes. Additionally, TNFα and IL-6 enhance PAI-1 and TF in adipose tissue, further exacerbating the procoagulant state, insulin resistance, and MCP-1 production from adipocytes and endothelial cells in adipose tissue [7].

Our study found a parallel increase in serum PAI-1 levels, serum TAG levels, hepatic TAG accumulation, and VAT mass. Interestingly, compared to the controls, the HHTg rat strain showed decreased serum and hepatic cholesterol concentrations that could be associated with the limited lipid capacity, especially for transport. Previous research has established a direct relationship between PAI-1 and VAT mass. Our findings are consistent with a clinical study that observed a positive correlation between PAI-1 levels and serum TAG levels [26]. Our study showed that, in severe hypertriglyceridemia, elevated circulating PAI-1 levels were accompanied by increased PAI-1 gene expression in VAT and PVAT, but not in hepatic tissue. Systematic inflammation induces an increase in PAI-1 gene expression in adipose tissue, followed by an elevation in serum PAI-1 levels. Therefore, both adipose tissue insulin resistance and hyperinsulinemia may contribute to increased PAI-1 secretion, consistent with our results. A study focusing on human adipocytes also showed that insulin increases PAI-1 [7].

Our study revealed increased PAI-1 gene expression in both systemically acting VAT and locally acting PVAT in prediabetic animals. Pathological processes in PVAT can contribute to impaired vascular function [34,35], while its vasoactive and metabolic activity may have a bearing on sex differences in vascular dysfunction. To our knowledge, this is the first study to investigate PAI-1 gene expression in PVAT in diabetic or prediabetic models.

In our previous study [11], HHTg females exhibited protective vasoactive mechanisms associated with milder hypertension, cardiac protection, and improved vascular function, despite the negative influence of the cyclooxygenase pathway. Additionally, preserved endothelial function and contractility were associated with PVAT activity. In the present study, increased PAI-1 in PVAT in HHTg rats was accompanied by the reduced expression of proinflammatory MCP-1 and IL-6 in PVAT. The reduced expression of these proinflammatory markers probably reflects their manner of downregulation by their increased circulating levels, thus supporting the protective effects of PVAT [11]. Therefore, PAI-1 could represent a potential therapeutic target in the early stages of vascular damage associated with severe dyslipidemia and insulin resistance. The tendency to show increased PAI-1 gene expression in the aorta in females (but not significantly) supports the hypothesis of higher PAI-1 activity in women, but this effect was not observed in other tissues.

PAI-1 dysregulation may be connected with hepatic lipid accumulation and fatty liver development, an independent cardiovascular risk factor. In obese patients with type 2 diabetes, increased liver fat was associated with elevated adipose tissue PAI-1 levels [36]. Thus, adipose-tissue-derived PAI-1 can contribute to the acceleration of fatty liver disease. A meta-analysis of 10,540 multiracial subjects found a significant association between serum PAI-1 levels and biopsy-proven or CT-diagnosed nonalcoholic fatty liver disease (NAFLD) [37].

Our study revealed an increase in the expression of adhesion molecules in the aortas of both male and female HHTg rats, which can lead to endothelial dysfunction. However, we observed a decrease in inflammatory TNFα in the aortas of HHTg females. Reduced aortic inflammation may contribute to a lower cardiovascular risk in females, despite their more pronounced dyslipidemia and ectopic triglyceride accumulation in the liver.

Vascular function can be affected by both inflammation and sex hormones. Estrogens have positive effects on lipid and lipoprotein metabolism, e.g., by increasing HDL-cholesterol [38]. In addition, sex hormones can affect the metabolic and inflammatory activity of VAT and, as recently shown, PVAT. Females show better anti-inflammatory and anti-contractility effects of PVAT; however, there are only few studies to date [12]. An increase in nuclear factor-kappa B (NF-κB), a transcription factor involved in inflammation-induced vascular impairment, can induce inflammatory cytokine production, leukocyte-attracting chemokines, and the expression of cell adhesion molecules [39,40]. Alterations in endothelial cells and monocytes can increase the synthesis of TF, a primary procoagulant found in atherosclerotic plaque, as well as PLT activation and aggregation, and changes in coagulation and fibrinolytic factors [31]. It has been shown that estrogens can increase platelet reactivity and determine the upregulation of both the prothrombotic and the proinflammatory functions of platelets [41]. On the other hand, testosterone can reduce IL-6 levels [42].

Our study found that prediabetic females exhibited more pronounced dyslipidemia, hepatic lipid accumulation, and worsened procoagulation markers compared to prediabetic males, despite less pronounced inflammation in the circulation, VAT, and the aorta. Sex-dependent differences in proinflammatory adipokines may contribute to these sex differences in cardiovascular impairment.

Though our study did not reveal any significant sex differences in PAI-1 concentration or tissue gene expression, there was a clear trend toward increased PAI-1 in the serum and aortas of HHTg females compared to males. A previous study of diabetic mice found higher PAI-1 activity in females than in males [43], which could be related to differences in VAT accumulation. Under diabetic conditions, women experience a greater relative increase in cardiovascular disease morbidity and mortality compared to men [2]. However, our results suggest that females, even in a prediabetic state, can maintain a lower cardiovascular risk according to the Framingham risk score. Less pronounced inflammation and better bioavailability of NO in prediabetic females may contribute to reduced cardiovascular impairment. It has been shown that a higher estrogen concentration has vasoprotective and anti-inflammatory effects [44].

Our previous study with the same rat strain [11] revealed that female prediabetic rats are more protected from vascular dysfunction than males, despite having more pronounced dyslipidemia. The milder hypertension and more efficient glucose utilization exhibited by prediabetic females may also be a contributing factor. Therefore, the interplay between inflammation, PAI-1, and the metabolic activity of PVAT may contribute to sex differences in cardiovascular risk and impairment, making them potential therapeutic targets. However, more studies are needed.

## 5. Conclusions

In summary, our results suggest that severe dyslipidemia and hepatic triglyceride accumulation in a prediabetic rat model was associated with hypercoagulation and the presence of low-grade chronic inflammation, with each sex contributing differently: males had particularly elevated leptin, while females had elevated interleukin 6. Regardless of their more pronounced dyslipidemia and procoagulation status, HHTg females showed less pronounced inflammation in the visceral adipose tissue, as well as in the aorta, compared to males, which could reduce the cardiovascular damage associated with prediabetes in females. On the other hand, increased PAI-1 in visceral and perivascular adipose tissues, together with an increased gene expression of ICAM in the aorta, in both sexes in the HHTg rat strain can indicate a link between inflammation and vascular dysfunction.

## Figures and Tables

**Table 1:** Basal metabolic characteristics.

	WKY Male	WKY Female	HHTg Male	HHTg Female	*p*-_STRAIN_	*p*-_SEX_	*p-*_INT_
Body weight	375 ± 4	251 ± 3 ***	432 ± 7	257 ± 2 ***	<0.001	<0.001	<0.001
Adiposity index	2.00 ± 0.10	1.96 ± 0.12 **	4.16 ± 0.10	4.74 ± 0.17 **	<0.001	<0.05	<0.05
Left ventricle weight	0.165 ± 0.004	0.169 ± 0.009	0.116 ± 0.004	0.148 ± 0.004 ***	<0.001	<0.001	<0.001
Fasting glucose	5.6 ± 0.1	5.5 ± 0.2	6.8 ± 0.1	6.5 ± 0.3	<0.001	n.s.	n.s.
Non-fasting glucose	9.09 ± 0.37	8.08 ± 0.25	10.46 ± 0.64	8.51 ± 0.70 *	<0.05	<0.01	n.s.
Insulin	0.236 ± 0.032	0.167 ± 0.029	0.372 ± 0.059	0.233 ± 0.068 *	<0.05	<0.05	n.s.
Serum TAG	0.64 ± 0.06	0.66 ± 0.07	4.19 ± 0.30	5.41 ± 0.38 **	<0.001	<0.05	<0.05
Serum cholesterol	2.35 ± 0.03	2.81 ± 0.08	1.71 ± 0.04	1.48 ± 0.08 *	<0.001	n.s.	n.s.
HDL-cholesterol	0.73 ± 0.01	0.83 ± 0.03	0.57 ± 0.01	0.46 ± 0.02 ***	<0.01	n.s.	n.s.
HOMA-IR	2.01 ± 0.08	1.84 ± 0.08	2.44 ± 0.09	2.29 ± 0.03	<0.001	<0.05	n.s.

Values are given as the mean ± SEM; *n* = 8 for each group; *p* reflects the probability of the strain effects analyzed using two-way ANOVA and Fisher’s LSD post hoc test. Two-way ANOVA results: *p*-_STRAIN_ denotes the significance of the strain effect (WKY vs. HHTg); *p*-_SEX_ denotes the significance of the sex effect; and *p*-_INT_ denotes the significance of the strain-by-sex interaction. For multiple comparisons, Fisher’s LSD post hoc test was used. * *p* < 0.05; ** *p* < 0.01; *** *p* < 0.001. WKY—Wistar–Kyoto rats; HHTg—hereditary hypertriglyceridemic rats; HOMA-IR—homeostatic model assessment of insulin resistance; n.s.—nonsignificant. Body weight (g); adiposity index and left ventricle weight (g/100 g); glucose, TAG, cholesterol, and HDL cholesterol (mmol/L); insulin (nmol/L).

**Table 2:** Hematological and coagulation factors.

	WKY Male	WKY Female	HHTg Male	HHTg Female	*p*-_STRAIN_	*p*-_SEX_	*p*-_INT_
Hb (g/L)	141 ± 1	129 ± 1 ***	142 ± 2	137 ± 2 *	<0.01	<0.001	<0.05
HCT (L/L)	0.44 ± 0.01	0.40 ± 0.01 **	0.45 ± 0.01	0.42 ± 0.01 *	n.s.	<0.01	n.s.
RBC (×10^12^/L)	8.77 ± 0.08	7.61 ± 0.10 ***	8.50 ± 0.07	7.50 ± 0.10 ***	<0.05	<0.001	n.s.
MCV (fL)	50.6 ± 0.1	52.8 ± 0.2 ***	52.4 ± 0.2	55.6 ± 0.1 ***	<0.001	<0.001	<0.01
MCH (pg)	16.0 ± 0.1	17.0 ± 0.1 ***	16.7 ± 0.1	18.2 ± 0.1 ***	<0.001	<0.001	<0.05
MCHC (g/L)	316 ± 1	322 ± 1 ***	320 ± 1	328 ± 1 ***	<0.001	<0.001	n.s.
RDW (%)	17.9 ± 0.1	13.8 ± 0.1 ***	17.3 ± 0.1	13.8 ± 0.1 ***	<0.01	<0.001	<0.01
Fibrinogen (g/L)	1.97 ± 0.05	1.71 ± 0.03 ***	2.10 ± 0.04	1.64 ± 0.05 ***	n.s.	<0.001	<0.05
Factor VIII (%)	602 ± 56	632 ± 26	556 ± 39	560 ± 55	n.s.	n.s.	n.s.
vWf (%)	84.8 ± 2.2	67.8 ± 1.7 ***	72.9 ± 1.8	58.5 ± 4.7 **	<0.01	<0.001	n.s.
AT (%)	119 ± 3	143 ± 4 ***	107 ± 2	111 ± 1	<0.001	<0.001	<0.01
PLT (×10^9^/L)	653 ± 10	701 ± 16	867 ± 11	924 ± 28 *	<0.001	<0.01	n.s.
MPV (fL)	7.31 ± 0.03	7.37 ± 0.03	7.79 ± 0.04	7.75 ± 0.03	<0.001	n.s.	n.s.
PCT (mL/L)	4.35 ± 0.06	4.50 ± 0.10	6.56 ± 0.14	6.53 ± 0.18	<0.001	n.s.	n.s.

Values are given as the mean ± SEM; *n* = 8 for each group; *p* reflects the probability of the strain effects analyzed using two-way ANOVA and Fisher’s LSD post hoc test. Two-way ANOVA results: *p*-_STRAIN_ denotes the significance of the strain effect (WKY vs. HHTg); *p*-_SEX_ denotes the significance of the sex effect; and *p*-_INT_ denotes the significance of the strain-by-sex interaction. For multiple comparisons, Fisher’s LSD post hoc test was used. * *p* < 0.05; ** *p* < 0.01; *** *p* < 0.001. WKY—Wistar–Kyoto rats; HHTg—hereditary hypertriglyceridemic rats, n.s.—nonsignificant.

## Data Availability

All data arising from this study are contained within the article.

## Abbreviations

AT	antithrombin
Hb	hemoglobin
HCT	hematocrit
HOMA-IR	homeostatic model assessment of insulin resistance
hsCRP	high-sensitivity C-reactive protein
ICAM-1	intercellular adhesion molecule-1
IL-6	interleukin-6
IRS-1	insulin receptor substrate-1
LPL	lipoprotein lipase
MCP-1	monocyte chemoattractant protein-1
MCH	mean concentration of hemoglobin
MCHC	mean corpuscular hemoglobin concentration
MCV	mean corpuscular volume
MPV	mean platelet volume
NAFLD	nonalcoholic fatty liver disease
NF-κB	nuclear factor-kappa B
NO	nitric oxide
NOS	nitric oxide synthase
PAI-1	plasminogen activator inhibitor-1
PCT	plateletcrit
PLT	platelet
PVAT	perivascular adipose tissue
RBC	red blood cell
RDW	red blood cell distribution width
SREBP1	sterol regulatory element-binding protein 1
TAG	triglyceride
TF	tissue factor
tPA	tissue plasminogen activator
TNFα	tumor necrosis factor alpha
VAT	visceral adipose tissue
vWf	von Willebrand factor
WBC	white blood cell
